# Case report: Nocturnal low-frequency stimulation of the centromedian thalamic nucleus improves sleep quality and seizure control

**DOI:** 10.3389/fnhum.2024.1392100

**Published:** 2024-06-05

**Authors:** Surya Suresh, Ganne Chaitanya, Ajay Deep Kachhvah, Vladimir Vashin, Manojkumar Saranathan, Sandipan Pati

**Affiliations:** ^1^Department of Neurology, Texas Institute of Restorative Neurotechnologies, The University of Texas Health Science Center at Houston, Houston, TX, United States; ^2^Department of Radiology, University of Massachusetts Chan Medical School, Worcester, MA, United States

**Keywords:** DBS, centromedian nucleus, EEG, sleep, epilepsy

## Abstract

Sleep disturbances and drug-resistant seizures significantly impact people with idiopathic generalized epilepsy (IGE). Thalamic deep brain stimulation (DBS) offers potential treatment, but its effect on sleep and seizure control needs clarification. In this study, we combined wearable sleep monitoring with electroencephalogram (EEG) confirmation to investigate the impact of nocturnal centromedian nucleus (CM) DBS parameters in a patient with drug-resistant IGE. We found that high-frequency (125 Hz) CM stimulation during sleep severely disrupted sleep macro architecture and exacerbated seizures. Conversely, switching to low-frequency (10 Hz) stimulation enhanced both sleep quality and seizure control. This study underscores the critical need to personalize DBS settings, tailoring them to individual patients’ sleep patterns to maximize therapeutic benefits. While larger-scale trials are needed, our findings pave the way for patient-centric approaches to thalamic neuromodulation, offering a transformative path to improve treatment outcomes and quality of life for those with refractory epilepsy.

## Introduction

The relationship between sleep and seizures is intricate and bidirectional, with sleep stages influencing seizure thresholds and disrupted sleep potentially exacerbating seizure susceptibility (Moore et al., 2021; Lehner et al., 2022; Nobili et al., 2022). This phenomenon is particularly significant in Idiopathic Generalized Epilepsy (IGE), characterized by generalized spike–wave paroxysms regulated by the thalamo-cortico-reticular system. Studies involving feline epilepsy models and IGE patients have unveiled a compelling pattern, wherein spike–wave epileptiform paroxysms peak during non-rapid eye movement (NREM) sleep stages, diminish during brief arousals, and abruptly decrease during rapid eye movement (REM) sleep (Halász and Dévényi, 1974; Kellaway et al., 1980; Kostopoulos et al., 1981a,b). Notably, Janz identified two peaks of increased seizures: one during the transition from wakefulness to sleep (post-meal peak) between 9 and 11 PM and another during the transition from sleep to wakefulness between 3 and 5 AM (Janz, 2000). Gloor’s corticoreticular hypothesis reconciled the thalamocortical and corticothalamic origins of generalized spike–waves, illustrating the transformation of spindles into spike–wave complexes during sleep onset (Gloor, 1968). The thalamic reticular nucleus, along with the intralaminar thalamus connected to the brainstem reticular system, plays a pivotal role in modulating generalized spike–wave discharges in IGE, highlighting the critical role of sleep in modulating seizure susceptibility in these patients (Gloor, 1968; Tucker et al., 2009). Steriade et al. highlighted the relationship between slow-wave oscillations during NREM sleep and the spontaneous spike–wave paroxysms observed in generalized epilepsies. Experimental studies have suggested that spike–wave discharges of IGE represent the epileptic exaggeration of the bursting mode of the thalamocortical system, with the spike resembling slow-wave sleep “UP” state while the waves of spike–waves resembling the “DOWN” state of slow-wave sleep (Tucker et al., 2009).

The centromedian nucleus of the thalamus (CM), a vital component of the intralaminar thalamus, plays a crucial role in regulating both arousal and sleep–wake cycles and represents a prime target for deep brain stimulation (DBS) in cases of drug-resistant IGE (Ilyas et al., 2019; Cukiert et al., 2020; Ubaid Hafeez et al., 2023). In clinical practice, high-frequency (>100 Hz) continuous CM stimulation is customary, inducing arousal (Martin et al., 2021). However, despite patient reports of sleep disturbances, its systematic impact on sleep remains unexplored, leaving unanswered questions regarding the optimal thalamic stimulation approach for preserving sleep microarchitecture while controlling seizures.

In this case report, we aimed to investigate changes in sleep macroarchitecture across multiple nights by varying nocturnal (restricted between 9 PM-6 AM) CM stimulation parameters, comparing low-frequency (10 Hz) stimulation with high-frequency (125 Hz) stimulation.

We hypothesized that high frequency stimulation of the centromedian thalamus is likely to affect sleep and seizure outcome in a more disruptive manner compared to low-frequency (10 Hz) stimulation. Prior studies with stimulation of different thalamic nuclei like anterior thalamus (ANT), ventral intermediate (VIM) have shown reduced total sleep times and increase in percentage of N2 increased and wakefulness after sleep onset (WASO) (Velasco et al., 2000; Arnal et al., 2020).

This study seeks to illuminate the intricate relationship between thalamic neuromodulation, sleep quality, and seizure control, potentially offering valuable insights to enhance patient outcomes.

## Method

### Case presentation

A 39-year-old right-handed male, with history of hypertension, obesity, prior COVID pneumonia, diagnosed with drug-resistant IGE (Juvenile Myoclonic Epilepsy) at 14y of age presented to an outside hospital with status epilepticus. He reported experiencing multiple myoclonic jerks, daily 15–20 brief (<10 s) absence seizures, and one bilateral tonic–clonic seizure (BTCS) every 2–3 years. Despite treatment with medications including valproate, brivaracetam, lamotrigine, and clobazam, and an unsuccessful trial of vagus nerve stimulation, his seizures remained uncontrolled. Video EEG evaluation showed generalized 3 Hz spike–wave discharges noted, frequently occurring in runs of 10–12 s, clinically associated with myoclonus consistently and occasionally with eye blinks. At that time, he was noted to have 1–2 episodes per hour, clinically lasting 3–4 s. 3 Tesla MRI brain was normal. Due to the intractable and generalized nature of his seizures, a decision was made to implant a deep brain stimulator (Medtronic, Percept, Minnesota) in bilateral centromedian nucleus of the Thalamus (CM) (Velasco et al., 2000). Following a six-weeks observation post-implantation, DBS was activated. Initially, continuous stimulation at 125 Hz, 90 microseconds, with current 3 mA was employed and the patient reported sleep disturbances.

**Figure 1:**
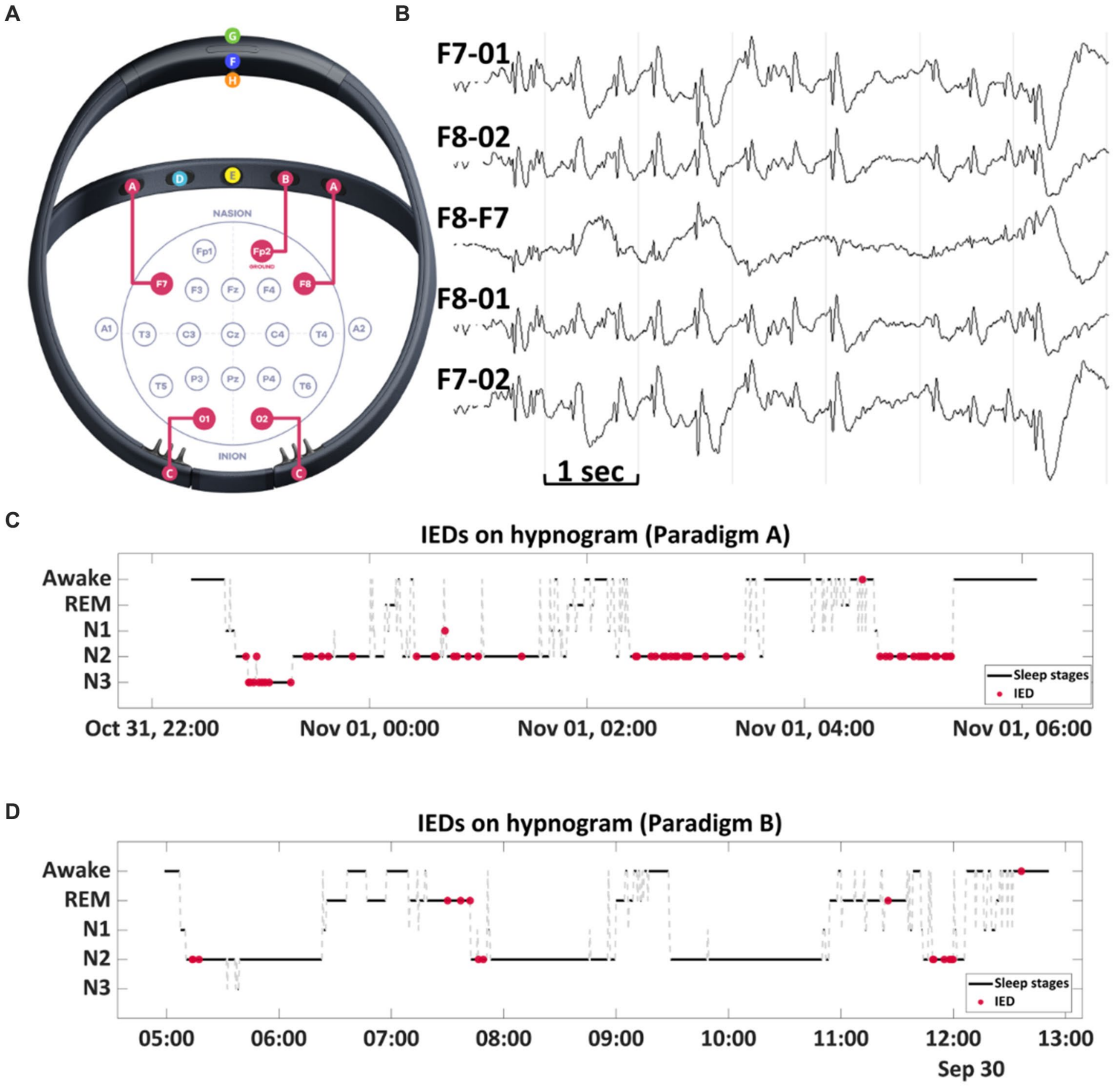
**(A)** Dreem 3 EEG headband. The EEG system comprises five electrodes: two frontal sensors [A] located at F7 and F8 positions, a ground sensor [B] positioned on the frontal band at Fp2, and two occipital sensors [C] placed at O1 and O2 positions. The device is equipped with an R&D sensor [D] for pulse oximetry and an audio output by a bone conduction speaker [E]. An accelerometer [F] is integrated to gauge movements, head position, and respiratory rate. Other features include a power button [G] for device control and a magnet port [H] for charging. **(B)** A sample EEG recording from Dreem 3 displays spike and wave complexes. **(C,D)** The RED dots show the temporal distribution of interictal discharges, and they are represented on the hypnogram generated by the headband. A higher occurrence of interictal discharges was noted during high-frequency stimulation compared to low-frequency stimulation.

This prompted enrollment in an Institutional Review Board (IRB) - approved investigation utilizing the Dreem 3 headband (DH) to confirm and quantify sleep disturbances (Figure 1). The DH, a soft, flexible wearable headband equipped with five dry EEG electrodes (O1, O2, FpZ, F7, and F8), a 3D accelerometer, and an infra-red pulse oximeter, records and automatically analyzes physiological signals in real time. The sampling frequency of the device is 250 Hz. The DH device records, stores, and automatically analyzes physiological signals in real time without requiring any Bluetooth connections (Arnal et al., 2020; Chouraki et al., 2023). The device employs a validated deep-learning algorithm for automatic sleep staging.

The patient wore the headband during sleep for over 15 days, distributed throughout the month, while maintaining DBS continuous stimulation at 125 Hz both day and night. Data from the DH confirmed sleep disturbances, leading to an offer of alternative low-frequency (10 Hz) stimulation at night (stimulation paradigm B).

### Stimulation paradigm

**Figure 2:**
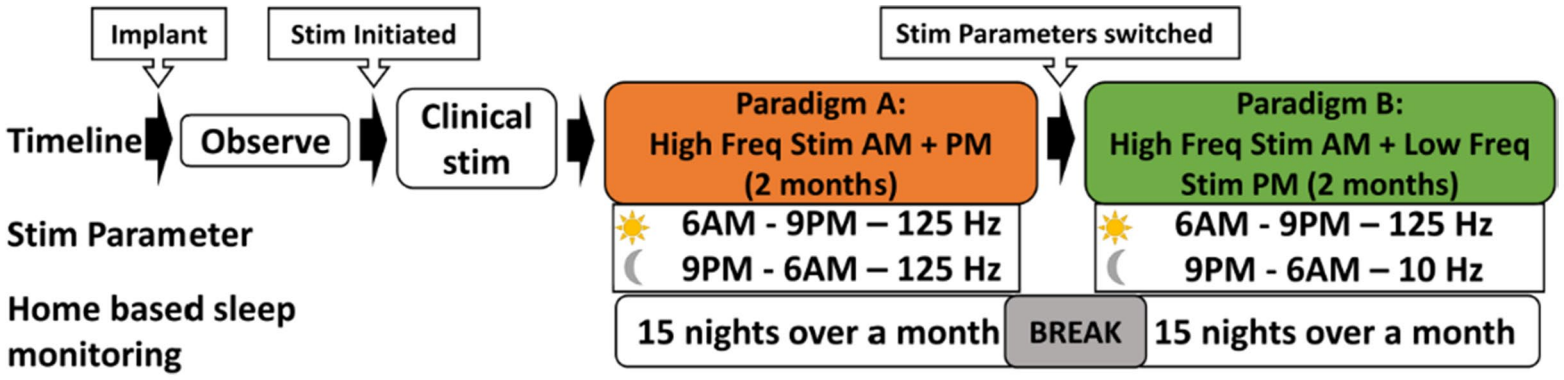
N-of-1 Study Design: this cross-over experimental design aimed at assessing the impact of two different stimulation parameters on sleep patterns. Following implantation, patient is given an observation period of 6 weeks. Following that, the participant received continuous stimulation at 125 Hz. Subsequently, the stimulation parameters are adjusted to two settings: 125 Hz during 6 AM to 9 PM, and 10 Hz during 9 PM to 6 AM, over a subsequent two-month period. Dreem 3 device was used to obtained home-based sleep recordings during both these stimulation paradigms. Each stimulation paradigm was tested for 15 nights, interspersed with a 3-night break period, resulting in a total of 30 sleep recordings.

The crossover paradigm involved two stimulation paradigms. In Paradigm A (Nocturnal high frequency stimulation), continuous stimulation at 125 Hz (pulse width: 90 microsecond and amplitude: 3 mA) was administered both day and night for 2 months. Then the stimulation was switched to Paradigm B (Nocturnal low frequency stimulation), wherein stimulation was delivered at 125 Hz 90 microseconds pulse width and 3 mA intensity from 6 AM to 9 PM, while continuous stimulation at 10 Hz with 90 microseconds pulse width and 3 mA intensity was applied from 9 PM to 6 AM (Figure 2). The patient is advised to adhere to a consistent schedule for switching the stimulation settings, ideally before going to bed around 9 PM and upon waking up around 6 AM. This ensures that the patient receives high frequency stimulation during the daytime and low frequency at night. The numbering is 0 to 3 on the right and 8–11 on the left. On the right side the anode is contact number 1, and the cathode is contact number 2. On the left side the anode is contact number 9, and the cathode is contact number 10.

### Analysis

Quantification of interictal discharges- Fragments of generalized spike–wave discharges were observed in the bipolar montage EEG channels recorded with DH. A physician trained in EEG interpretation counted the number of spike–wave discharges recorded during sleep for 15 nights with low-frequency stimulation and 15 nights with high-frequency stimulation using visual inspection. Analysis was restricted to artifact-free EEG.

### Sleep metrics

Sleep metrics available from the DH device include total sleep time (TST), sleep efficiency, wakefulness after sleep onset (WASO), and sleep stages N1, N2, N3, and REM. Sleep metrics for five nights per stimulation parameter (low and high-frequency) were analyzed for statistical significance.

### DBS electrode localization

**Figure 3:**
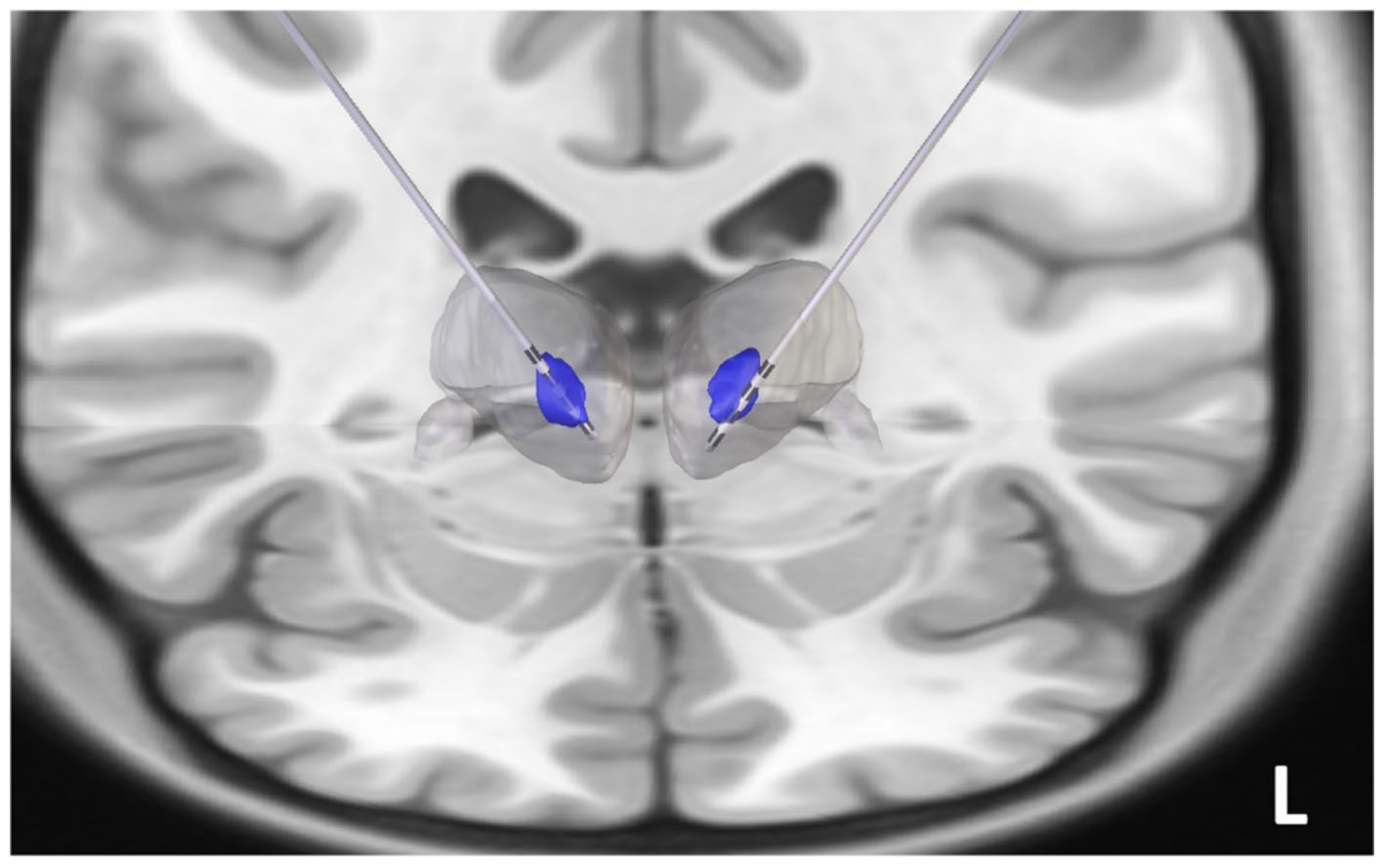
Reconstruction of the centromedian (CM) thalamic deep brain stimulation (DBS) electrodes: The post-implant CT scan was coregistered to pre-operative 3 T FGATIR images (Fast Gray Matter Acquisition T1 Inversion Recovery). Thalamus Optimized Multi Atlas Segmentation (THOMAS) segmentation algorithm was used to generate patient specific thalamic nucleic segmentation (blue regions represent the bilateral CM nuclei, the gray blob represents the thalamic boundary). DBS electrodes (Medtronic 3,389) were noted to be implanted in the CM thalamus.

Similar to our prior studies (Vakilna et al., 2023), Thalamus Optimized Multi-Atlas Segmentation (THOMAS) was utilized on the preoperative Fast Gray Matter Acquisition T1 Inversion Recovery (FGATIR) sequence to create the patient’s own CM nucleus segmentation (Su et al., 2019; Vakilna et al., 2023). Post-implant CT and the FGATIR sequences were coregistered to the pre-implant T1 MRI using the Advanced Normalization Tools (ANTS). The segmented CM nucleus was overlaid on the structural T1, and RNS leads were reconstructed in LeadDBS to determine if the electrodes were localized in the CM (Horn et al., 2019) (Figure 3).

### Statistical analysis

Descriptive statistics were displayed as mean ± standard deviation (SD). A paired t-test was conducted to calculate the significant difference in interictal discharges between low-frequency and high-frequency stimulation during sleep. Similarly, paired *t*-tests were performed to compare the sleep metrics (WASO, N1-N3, REM, TST) for the two stimulation parameters. The level of statistical significance (*p* value) was set at 0.05. The percentage reduction in seizure was calculated as (Initial/baseline number - Final number) / (Initial/baseline number) X 100.

## Results

### Seizure outcome

Before DBS implantation, the patient had been experiencing 15–20 absence seizures daily, 20 myoclonic jerks per week with no reported generalized convulsions in the preceding years (3–5 BTCS in his lifetime). Over the two-month treatment period with Paradigm A, involving daytime and nocturnal high-frequency stimulation, the patient experienced 40% increase in daily absence seizures compared to the pre-implantation baseline. During Paradigm A, the patient continued to experience multiple seizures per day, along with 1 BTCS. In contrast, following 2 months of treatment with Paradigm B, utilizing nocturnal low-frequency stimulation, there was a notable improvement in outcomes. The patient reported nearly a 90% reduction in absence seizures and myoclonic jerks, decreasing from 20 seizures per week to 2–5 seizures per week, compared to both the baseline and the high-frequency stimulation period.

### Interictal burden

**Figure 4:**
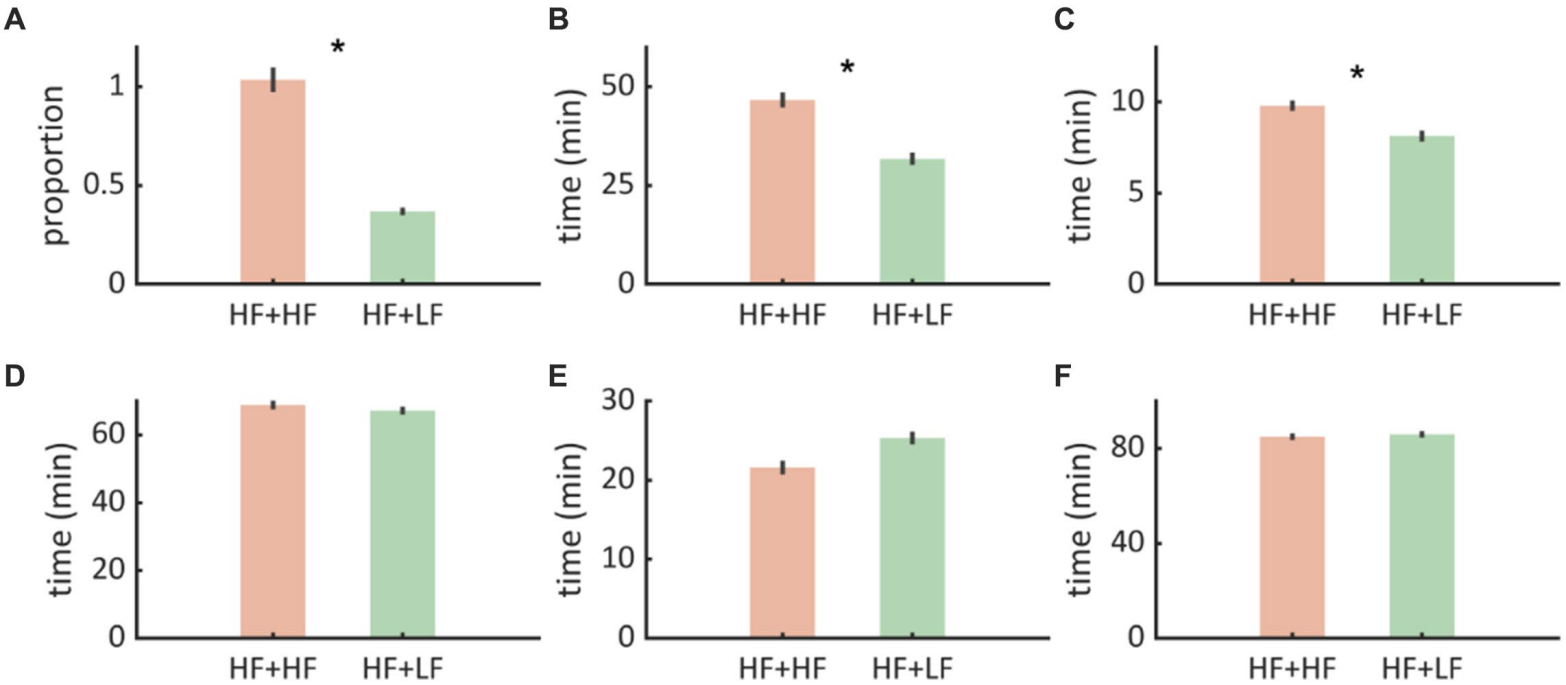
**(A)** We noted that Paradigm B (Nocturnal low frequency stimulation - HF + LF) was associated with proportionately fewer interictal discharges compared to Paradigm A (Nocturnal high frequency stimulation - HF + HF). **(B,C)** WASO and N1 durations were shorter with Paradigm B implying that there was less sleep disruption. **(D–F)** N2, N3, REM, and Sleep efficiency were not significantly affected. The error bars represent standard error of mean.

Interictal spike counts were estimated as a proportion of spikes/Total sleep time (TST) recorded by Dreem headband for 15 days. Paradigm A (nocturnal high frequency stimulation) was associated with a higher interictal burden compared to Paradigm B (nocturnal low frequency stimulation) (0.683 vs. 0.248, *z* = 2.3, *p* = 0.016) (Figure 4A).

### Sleep outcome

Among all sleep metrics, WASO was lower during Paradigm B (nocturnal low frequency stimulation) compared to Paradigm A (31.76 ± 14.49 vs. 46.63 ± 19.60, *t* = −2.36, *p* = 0.025), indicating a marked reduction in sleep fragmentation. Similarly, Stage N1 was shorter during Paradigm B (nocturnal low frequency stimulation) (8.13 ± 2.61 vs. 9.8 ± 2.27, *t* = −2.14, *p* = 0.02) compared to Paradigm B (nocturnal low frequency stimulation), indicating fewer arousals/transitions to early sleep stages. No differences were found in other sleep metrics (N2, N3, REM, and sleep efficiency) (Figures 4B–F).

## Discussion

Our findings reveal that high-frequency stimulation of the CM thalamus can disrupt sleep macroarchitecture and worsen seizure control in a patient with drug-resistant IGE. Conversely, switching to low-frequency stimulation significantly improved both sleep quality and seizure control. These results highlight the intricate relationship between thalamic neuromodulation, sleep quality, and seizure control, underscoring the importance of optimizing stimulation parameters for improved clinical outcomes.

The pathophysiology of generalized spike–wave discharges and seizures in Idiopathic Generalized Epilepsy (IGE) has long been linked to sleep, dating back to Gowers’s observations in 1885 (Gowers et al., 1885). In IGE, epileptiform paroxysms are more prominent during NREM sleep stages, possibly due to the transformation of NREM sleep spindles into spike–wave discharges in hyperexcitable cortical states. While sleep facilitates epileptiform discharge formation, sleep deprivation is also a potent trigger for epileptic seizures. Klingler et al. (1991) demonstrated higher epileptiform discharges in waking EEGs after sleep deprivation compared to sleep EEGs after sleep deprivation. Chronic sleep disruption may thus increase epileptiform discharge burden, as observed in our case study.

Wake after sleep onset (WASO) measures wakefulness periods after sleep onset, reflecting poor sleep quality and increased sleep fragmentation (Shrivastava et al., 2014). High-frequency stimulation significantly increased WASO and N1 duration compared to low-frequency stimulation, suggesting possible modulation of the arousal network. The CM thalamus, a component of the intralaminar thalamus connected to the reticular activating system, has consistently been demonstrated to induce arousal with high-frequency stimulation (Martin et al., 2021). The CM neurons play a central role in dual control, managing cortical up-down states during NREM sleep as well as arousal and consciousness (Gent et al., 2018). Notably, optogenetic tonic activation of CM neurons consistently induced rapid awakening from NREM sleep when optical stimulations exceeded 500 ms in duration. Conversely, stimulation mimicking spontaneous burst firing led to slow-wave-like activity, promoting enhanced cortical synchrony and sleep.

The impact of deep brain electrical stimulation frequencies on behavioral and cortical responses hinges on the stimuli’s selective recruitment and engagement of different neural elements (McIntyre and Grill, 2002). An experimental study has revealed that higher frequencies (>100 Hz) can activate various cortical regions, such as the frontal cortex, motor cortex, somatosensory cortex, and striatum, all receiving extensive glutamatergic inputs from the intralaminar nuclei (Liu et al., 2015). Consequently, high-frequency thalamic stimulation during sleep in our patient may have inadvertently triggered widespread cortical activation associated with arousal. Conversely, 10 Hz stimulation during sleep likely limited forebrain activation and prevented disrupting the generation of slow-wave oscillations, as evidenced by experimental findings (Liu et al., 2015).

The stimulation frequency for the anterior nucleus of the thalamus in the SANTE trial was 140 Hz with 1 min on and 5 min off (Fisher et al., 2010). Although a sleep study was not performed in that study, stimulation-induced sleep disturbances secondary to arousal were later reported in nine patients (Voges et al., 2015).

Prior research by Mivalt et al. (2022) integrated polysomnography and intracranial electroencephalography alongside both low- and high-frequency anterior nucleus of thalamus DBS through an innovative implantable neural device. The strengths of our study include the within-individual trial design, which partly controls for changes in sleep–wake attributed to anti-seizure medications or other undiagnosed comorbidities. Another major strength is the innovative use of wearable headbands, which provide multi-night objective sleep data and EEG, confirming increased epileptiform paroxysms. However, the study is limited by its case-study nature, highlighting the need for larger-scale studies to evaluate changes in sleep with thalamic neuromodulation. In addition, the Dreem headband and the automated scoring have their limitations. (1) The limited EEG channels allow the detection of epileptic spikes and seizures in spatially selected brain regions, although this limitation may not be applicable in generalized epilepsies. (2) The data quality is dependent on patient compliance and wearing the headband in a specific way as directed. (3) The automated scoring had an overall accuracy of 83.5 ± 6.4% (F1 score: 83.8 ± 6.3) for the dreem headband in comparison with the expert scoring 86.4 ± 8.0% (F1 score: 86.3 ± 7.4) (Arnal et al., 2020).

In conclusion, this prospective crossover study offers valuable insights into the complex interplay between sleep, seizures, and thalamic neuromodulation. By systematically evaluating alterations in sleep macro architecture during multi-night home monitoring, we shed light on the impact of varying nocturnal stimulation parameters. This research represents a pioneering effort as the first study to employ multi-night home-based sleep monitoring to assess the impact of thalamic neuromodulation at different frequencies. Such an approach has the potential to offer novel insights and guide future studies aiming to enhance the quality of life for individuals with refractory epilepsy by fine-tuning thalamic stimulation parameters.

## Data availability statement

The raw data supporting the conclusions of this article will be made available by the authors, on request.

## Ethics statement

The studies involving humans were approved by the Committee for the Protection of Human Subjects (CPHS) is the name of the Institutional Review Board for the University of Texas Health Science Center at Houston. The studies were conducted in accordance with the local legislation and institutional requirements. The participants provided their written informed consent to participate in this study. Written informed consent was obtained from the individual(s) for the publication of any potentially identifiable images or data included in this article.

## Author contributions

SS: Conceptualization, Data curation, Formal analysis, Writing – original draft, Writing – review & editing. GC: Data curation, Formal analysis, Methodology, Supervision, Validation, Writing – review & editing. AK: Data curation, Formal analysis, Validation, Writing – review & editing. VV: Data curation, Formal analysis, Writing – review & editing. MS: Software, Writing – review & editing. SP: Conceptualization, Data curation, Investigation, Methodology, Project administration, Supervision, Validation, Writing – original draft, Writing – review & editing.
